# A novel method for assessment of airway opening pressure without the need for low-flow insufflation

**DOI:** 10.1186/s13054-023-04560-0

**Published:** 2023-07-07

**Authors:** Anne-Fleur Haudebourg, Elsa Moncomble, Arnaud Lesimple, Flora Delamaire, Bruno Louis, Armand Mekontso Dessap, Alain Mercat, Jean-Christophe Richard, François Beloncle, Guillaume Carteaux

**Affiliations:** 1 Assistance Publique-Hôpitaux de Paris, CHU Henri Mondor-Albert Chenevier, Service de Médecine Intensive Réanimation, 51, Avenue du Maréchal de Lattre de Tassigny, 94010 Créteil Cedex, France; 2 Groupe de Recherche Clinique CARMAS, Faculté de Santé, Université Paris Est-Créteil, 94010 Créteil, France; 3 INSERM U955, Institut Mondor de Recherche Biomédicale, 94010 Créteil, France; 4 CNRS, INSERM 1083, MITOVASC, Université d’Angers, Angers, France; 5 Laboratoire Med2Lab ALMS, Antony, France; 6 Département de Médecine Intensive-Réanimation et Médecine Hyperbare, Centre Hospitalier Universitaire d’Angers, Vent’ Lab, Faculté de Santé, Université d’Angers, Angers, France; 7 UMR 1066, INSERM, Créteil, France

**Keywords:** Mechanical ventilation, Respiratory mechanics, Airway opening pressure, Acute respiratory distress syndrome, Protective ventilation

## Abstract

**Background:**

Airway opening pressure (AOP) detection and measurement are essential for assessing respiratory mechanics and adapting ventilation. We propose a novel approach for AOP assessment during volume assist control ventilation at a usual constant-flow rate of 60 L/min.

**Objectives:**

To validate the conductive pressure (*P*_cond_) method, which compare the *P*_cond_—defined on the airway pressure waveform as the difference between the airway pressure level at which an abrupt change in slope occurs at the beginning of insufflation and PEEP—to resistive pressure for AOP detection and measurement, and to compare its respiratory and hemodynamic tolerance to the standard low-flow insufflation method.

**Methods:**

The proof-of-concept of the *P*_cond_ method was assessed on mechanical (lung simulator) and physiological (cadavers) bench models. Its diagnostic performance was evaluated in 213 patients, using the standard low-flow insufflation method as a reference. In 45 patients, the respiratory and hemodynamic tolerance of the *P*_cond_ method was compared with the standard low-flow method.

**Measurements and main results:**

Bench assessments validated the *P*_cond_ method proof-of-concept. Sensitivity and specificity of the *P*_cond_ method for AOP detection were 93% and 91%, respectively. AOP obtained by *P*_cond_ and standard low-flow methods strongly correlated (*r* = 0.84, *p* < 0.001). Changes in SpO_2_ were significantly lower during *P*_cond_ than during standard method (*p* < 0.001).

**Conclusion:**

Determination of *P*_cond_ during constant-flow assist control ventilation may permit to easily and safely detect and measure AOP.

**Supplementary Information:**

The online version contains supplementary material available at 10.1186/s13054-023-04560-0.

## Background

Airway closure phenomenon [1] has been reported in 23–52% patients with acute respiratory distress syndrome ARDS [2–9]. For such patients, the airways remain closed until the airway pressure reaches a specific threshold known as the airway opening pressure (AOP), beyond which the airways become open [10–12]. Thus, lung inflation begins when the airway pressure overcomes the AOP [13]. If neglected, this phenomenon may bias the assessment of respiratory mechanics when positive end-expiratory pressure (PEEP) is set below the AOP [3]. Cyclic opening and closing of small airways may also occur and promote ventilator-induced lung injury [14]. Therefore, it is important to look for a potential airway closure in ARDS in order to customize mechanical ventilation.

**Fig. 1:**
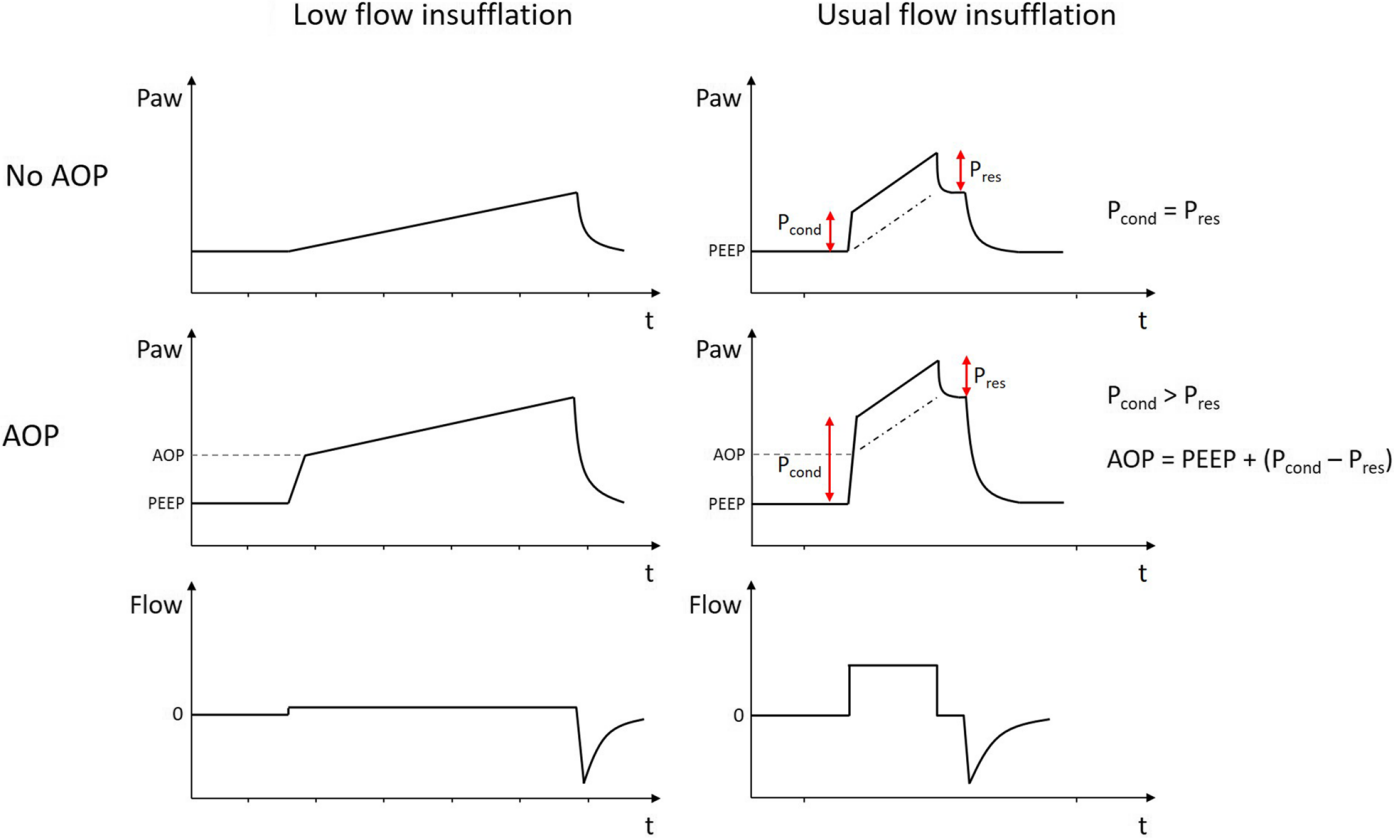
Principles of standard and new methods for the detection and measurement of airway opening pressure. Left: principle of the detection and measurement of airway opening pressure (AOP) according to the standard method. Using low-flow insufflation (5 L/min), AOP is detected as the presence of an abrupt change in slope on the time-pressure curve, with a first extremely low slope. The value of the airway pressure at the level of the slope change provided the value of the AOP. Right: principle of the detection and measurement of AOP according to the new method using the conductive pressure (*P*_cond_). During usual constant-flow volume assist control ventilation (e.g., with a flow rate of 60 L/min), the *P*_cond_ is identified on the airway pressure waveform as the difference between the abrupt change in slope at the very beginning of the insufflation and the PEEP. When *P*_cond_ is equal to the resistive pressure (*P*_res_), it means that there is no airway closure phenomenon (top). AOP is detected when *P*_cond_ is significantly higher than *P*_res_ (*P*_cond_ − *P*_res_ > 1 cm H_2_O, middle panel). The AOP value is therefore defined as: AOP = PEEP + (*P*_cond_ − *P*_res_)

The method usually used to detect airway closure and measure AOP during mechanical ventilation requires a low-flow insufflation (i.e., 5 L/min) [1, 8] to make the resistive component of airway pressure negligible. AOP is identified as the presence of an abrupt change in slope on the pressure–volume curve (if available) or on the time-pressure curve (Fig. 1), with the first slope representing the ventilator’s circuit compliance because the airways are closed. Limited data suggest that this maneuver may be poorly tolerated by certain patients due to the reduced minute ventilation required for low-flow insufflation, as well as the decrease in PEEP [15, 16].

During volume assist control ventilation with usual constant-flow (i.e*.*, with a flow rate of 30–60 L/min), an abrupt change in slope is observed on the airway pressure waveform at the beginning of inflation. The difference between the airway pressure level at which this change in slope occurs and PEEP mainly represents the resistive pressure (P_res_) [17], which could be easily calculated at the end of insufflation as the difference between peak and plateau pressure [18] (Fig. 1). In the case of airway closure, the airway pressure level at which the abrupt change in slope occurs should be shifted upward since it represents the *P*_res_ above the AOP (Fig. 1). The difference between the airway pressure level at which the change in slope occurs on the airway pressure waveform and PEEP is hereafter referred to as the “conductive pressure” (*P*_cond_), as it represents the pressure needed to conduct the inspiratory flow through the airways, regardless of the presence of an AOP above PEEP.

In this study, we hypothesized that 1—airway closure can be detected and AOP measured during ventilation at a usual constant-flow rate using the difference between *P*_cond_ and *P*_res_ [18]; 2—this simplified detection would result in better clinical tolerance than the current standard method using low-flow insufflation.

## Methods

### AOP measurements and definitions

Conductive pressure (*P*_cond_): During usual constant-flow volume assist control ventilation (i.e*.*, with a flow rate of 60 L/min), the *P*_cond_ was defined as the difference between the airway pressure level at which the abrupt change in slope occurs at the very beginning of the insufflation on the airway pressure waveform and PEEP (Fig. 1). According to the equation of motion of the respiratory system (Paw = PEEPtot + *P*_res_ + *P*_el_ = PEEPtot + Rrs·Flow + Ers·Volume; where Paw: airway pressure; PEEPtot: total PEEP; *P*_res_: resistive pressure; *P*_el_: elastic pressure; Rrs: respiratory system resistance; Ers: respiratory system elastance), the *P*_cond_ should approximate the *P*_res_ because at the time of the abrupt change in slope the elastic pressure is negligible [18] (Fig. 1). However, in the case of AOP above the PEEP, the flow is delivered in the airways above the AOP. Thus, the *P*_cond_ should reflect the AOP and *P*_res_, the latter of which being easily calculated at the end of insufflation (Fig. 1).

The following methods were assessed for airway closure detection and AOP measurement:

- “Standard method”: Using low-flow insufflation (5 L/min), airway closure was detected as the presence of an abrupt change in slope on the time-pressure curve (Fig. 1) [1, 8]. The value of the airway pressure at which the slope changes provided the value of the AOP. Two investigators (FB and AL) detected airway closure and measured AOP with the standard method, blinded to the results of the new method described below. If a significant difference (> 1 cm H_2_O) was observed between the assessments of the two investigators, a consensus was reached with the input of a third investigator (J-CR).
- “*P*_cond_ method”: Using a usual constant-flow rate (60 L/min) in volume assist control ventilation, the *P*_cond_ was identified by visual inspection of the airway pressure waveform. Resistive pressure was also detected as the difference between the peak and plateau pressures measured after at least 0.3 s of end-inspiratory occlusion [18]. Detection of an airway closure was defined as: *P*_cond_ − *P*_res_ > 1 cm H_2_O. The AOP value was defined as: AOP = PEEP + (*P*_cond_ − *P*_res_) (Fig. 1). Two investigators (A-FH and EM) detected and measured AOP with the *P*_cond_ method, blinded to the results of the standard method. If a significant difference (> 1 cm H_2_O) was observed between the assessments of the two investigators, a consensus was reached with the input of a third investigator (GC).

All flow and airway pressure curves were recorded using a pneumotachograph and a differential pressure transducer inserted between the Y piece of the ventilator circuit and the test lung inlet or endotracheal tube (bench study and DriVV cohort) or directly from the ventilator (PREMIER Cohort) and then stored in computer for offline analysis using Acqknowledge software (see Additional file 1 for details).

### Study design

Our study was carried out in three steps:

1. Evaluation of the proof-of-concept of the new AOP measurement principle (*P*_cond_ method) using both a mechanical and a physiological bench model;
2. Assessment of the performance of the new method for both detection of airway closure and measurement of AOP in two cohorts of ARDS patients;
3. Comparison of the clinical tolerance of the different AOP measurement methods in a prospective single-center observational study.

#### Proof-of-concept evaluation: Bench study

The principle of the proof-of-concept evaluation was to use bench models with an airway closure to assess the *P*_cond_ method at two levels of PEEP: one below and one equal to or above the AOP. Theoretically, when PEEP was set below the AOP, *P*_cond_ should be greater than *P*_res_ and their difference should estimate the AOP. When the PEEP was set at or above the AOP, the difference between *P*_cond_ and *P*_res_ should drop to zero.

##### Mechanical bench

We used an Active Servo Lung 5000 test lung (ASL5000®; IngMar Medical, Pittsburg, PA, USA) to simulate passive patients. First, we simulated a patient model with an airway opening pressure of 10 cm H_2_O (see Additional file 1 for details). Second, we simulated two controls: a first one without airway closure and a linear compliance, a second one without airway closure but with a nonlinear compliance, as described in some ARDS patients [19], with a lower inflection point at 10 cm H_2_O, a compliance below the lower inflection point of 20 mL/cm H_2_O, and a compliance above the lower inflection point of 40 mL/cm H_2_O. Airway resistance was set to 10 cm H_2_O/L/sec for all conditions.

Volume assist control ventilation with constant-flow was applied to the three models. Each AOP measurement method was assessed at a PEEP of 5 and 12 cm H_2_O.

##### Physiological bench

Two Thiel embalmed cadavers (TEC) intubated and mechanically ventilated, in whom an AOP of 9 and 10 cm H_2_O was detected using the standard method were used to assess the *P*_cond_ method. TEC are human corpses embalmed after a method described by Walter Thiel [20, 21], whose aspect is close to the living anatomy and with preserved elasticity and flexibility. Standard method and *P*_cond_ method were assessed at zero and 10 cm H_2_O of PEEP (see Additional file 1 for details).

#### Performance of the new method: physiological study

We assessed the accuracy and diagnostic performances of the new method for both detection of airway closure and measurement of AOP in two prospective observational cohorts (DriVV, approved by the “CPP Sud-Ouest et Outre Mer III” ethics committee, and PREMIER, approved by “CPP Sud-Est I” ethics committee) collecting detailed data on respiratory mechanics in patients under invasive mechanical ventilation (see Additional file 1 for details). In accordance with French law, non-opposition to participate in the study from patients or their next of kin was obtained prior to inclusion in each study. In both cohorts, airway pressure and flow waveforms were recorded during passive volume assist control ventilation during low-flow insufflation (5L/min) and at a constant-flow rate of 60 L/min, both at a PEEP of 5 cm H_2_O. We selected recordings in patients with no clinical detection of spontaneous respiratory effort and without detection of intrinsic PEEP by visual inspection of expiratory flow during ventilation with usual constant-flow rate. Standard method was used to detect and measure AOP during low-flow insufflation, and *P*_cond_ method was used during usual constant-flow rate as described above.

#### Evaluation of clinical tolerance

The tolerance of the different methods was assessed in one of the two prospective observational cohorts (DriVV) during which respiratory and hemodynamic parameters were collected at each ventilatory adjustment needed for application of standard and *P*_cond_ methods. Both the tidal volume and FiO_2_ were set by the attending physician and kept constant during the study. For the standard method, the following settings were used: flow rate of 5 L/min, PEEP of 5 cm H_2_O, and respiratory rate (RR) of 5 breaths/min. Depending on the ventilator used, efforts were made to reach these settings and then resume the initial ventilation after one low-flow cycle as quickly as possible to maximize the tolerance. Thus, whenever possible, all settings were preselected and validated at once. For *P*_cond_ method, the following settings were used: flow was maintained at 60 L/min, PEEP of 5 cm H_2_O, and RR of 20 breaths/min. If intrinsic PEEP was detected by visual inspection of the expiratory flow, the RR was further decreased until it disappeared. Between each maneuver, all ventilators’ settings were resumed as previously set by the attending physician until SpO_2_ returned to baseline. SpO_2_, RR, heart rate (HR), systolic, diastolic and mean blood pressure were collected at baseline. During each maneuver, the lowest SpO_2_, the lowest and the highest HR, the highest systolic blood pressure and the lowest mean blood pressure were collected.

### Endpoints

The proof-of-concept was considered valid if, for a given bench model, the AOP was detected by *P*_cond_ method when the PEEP level was set below the AOP value and was not detected when the PEEP level was set at or above the AOP value.

To assess the performance of *P*_cond_ method, both airway closure detection and AOP measurement were assessed. Airway closure detection was assessed using sensitivity, specificity and other standard formulas, as detailed below. For AOP measurements, the main endpoint was the correlation between AOP measured by the standard method and AOP measured by *P*_cond_ method. The agreement between methods was also assessed using the Bland and Altman plot.

With regard to the assessment of the tolerance of each method, the main endpoint was the minimal SpO_2_ recorded during each measurement and its corresponding ventilator setting adjustments compared to the SpO_2_ at baseline. We also compared the proportion of patients experiencing a SpO_2_ ≤ 88% during each measurement.

### Statistics

Data were analyzed using GraphPad Prism 8.0.1 (San Diego, CA, USA) and SPSS Base 29.0 statistical software package (SPSS, Chicago, IL). Continuous data were expressed as medians (25th–75th percentiles) and compared using the Mann–Whitney test for independent variables. For related variables, the Friedman test was initially performed to assess overall differences, followed by the Wilcoxon signed-rank test for pairwise comparisons. A Bonferroni correction was applied in case of multiple comparisons. Categorical variables, expressed as percentages, were evaluated using Chi-square or Fisher exact tests as appropriate. A *p* < 0.05 was considered significant. Standard formulas were used to calculate the sensitivity, specificity, positive predictive value, negative predictive value, positive likelihood ratio, negative likelihood ratio, diagnostic accuracy, and Youden index (see Additional file 1). Linear correlation analysis was performed to assess whether relationships existed between the standard and *P*_cond_ methods. Spearman correlation coefficients (r) and uncorrected *p* values are presented. Bland–Altman analyses were performed to evaluate agreement between *P*_cond_ and standard methods [22]. Using the Bland–Altman method, the mean differences between both measurements and the 95% limits of agreement, defined as the mean differences ± 1.96* standard deviation, were calculated.

## Results

### Proof-of-concept assessment

#### Mechanical bench

**Fig. 2:**
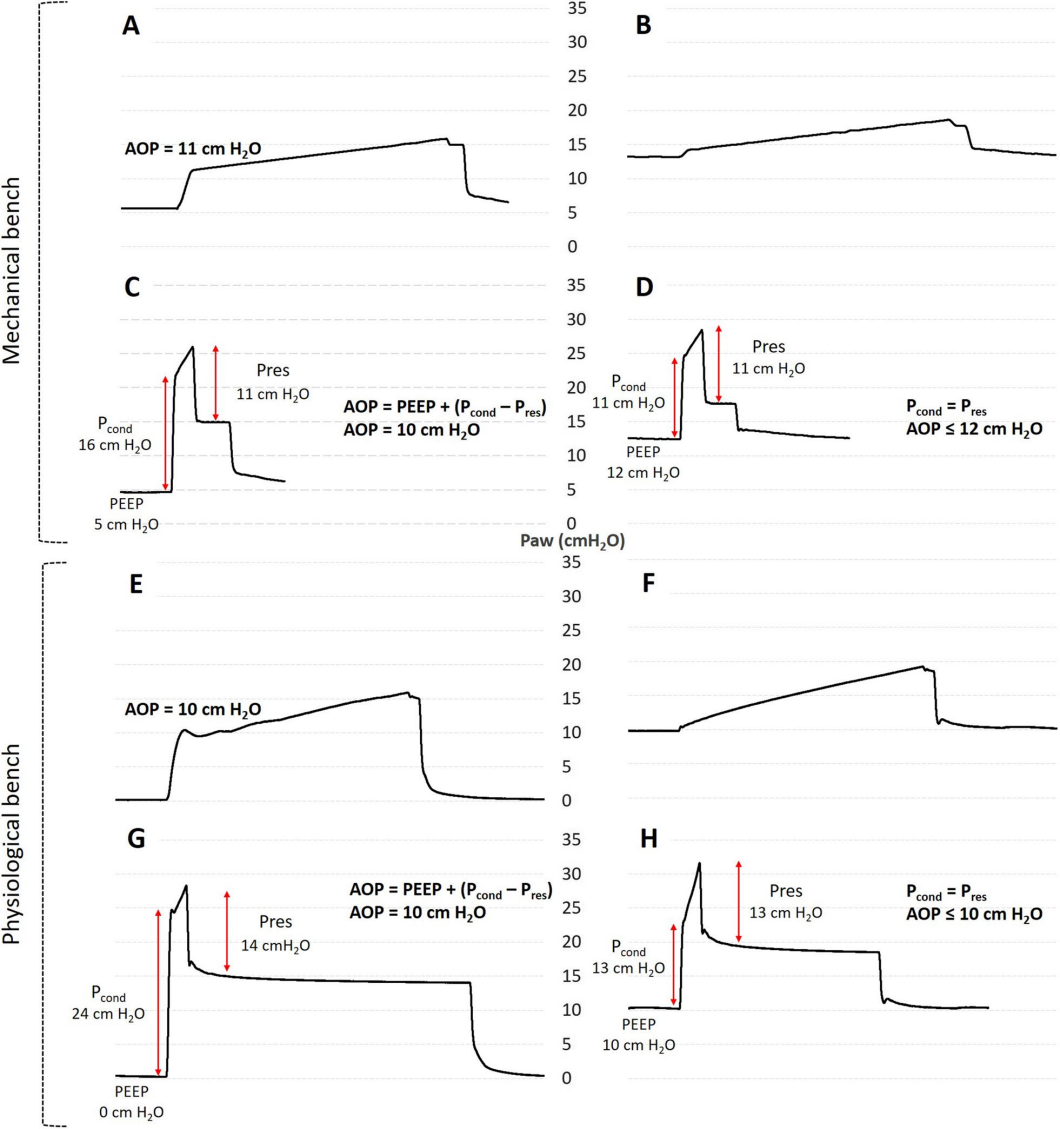
Proof-of-concept of the new method for airway opening pressure assessment on bench models. Each panel represents a time-airway pressure curve at low (**A**, **B**, **E**, **F**) or usual (**C**, **D**, **G**, **H**) constant-flow during assist control ventilation to assess airway opening pressure (AOP) by standard method and *P*_cond_ method, respectively. The left panels (**A**, **C**, **E**, **G**) represent experimental conditions where the PEEP is set below the AOP, and the right panels (**B**, **D**, **F**, **H**) conditions where the PEEP is set at or above the AOP. An AOP of 10 cm H_2_O was simulated for the mechanical bench (**A**–**D**). Recordings from one of the two Thiel embalmed cadavers used for the physiological bench are shown (**E**–**H**). Note that for each model, when the PEEP is set below the AOP, the conductive pressure (*P*_cond_) is greater than the resistive pressure (*P*_res_), and that the AOP can be calculated as: AOP = PEEP + (*P*_cond_ − *P*_res_). When the PEEP is set at or above the AOP, *P*_cond_ becomes equal to *P*_res_

Detection and measurement of AOP using the standard and *P*_cond_ methods according to the different bench models are reported in Table 1. When simulating an AOP of 10 cm H_2_O, the *P*_cond_ method actually detected an AOP of 10 at a PEEP of 5 cm H_2_O. When the PEEP was increased to 12 cm H_2_O, no AOP was detected above the new PEEP level with *P*_cond_ method, validating the proof-of-concept on the mechanical bench (Fig. 2).

**Table 1:** Airway opening pressure measurements with standard and P_cond_ methods according to mechanical bench models

	Method		Standard method	*P*_cond_ method
	Flow rate		5 L/min	60 L/min
AOP_sim_ = 10 cm H_2_O		PEEP 5 cm H_2_O	11	10
		PEEP 12 cm H_2_O	No AOP	No AOP
No airway closure	Control 1	PEEP 5 cm H_2_O	No AOP	No AOP
		PEEP 12 cm H_2_O	No AOP	No AOP
	Control 2	PEEP 5 cm H_2_O	No AOP	No AOP
		PEEP 12 cm H_2_O	No AOP	No AOP

AOP_sim_: simulated airway opening pressure; Control 1: simulated passive patient with linear compliance and no airway closure; Control 2: simulated passive patient with nonlinear compliance and no airway closure; *P*_cond_: conductive pressure (see text and Fig. 1 for definition); “No AOP” denotes no detection of AOP above the level of PEEP. Values of measured AOPs are given in cm H_2_O. See text and Fig. 1 for definitions of standard and *P*_cond_ methods

#### Physiological bench

The AOPs of the two TEC measured using the standard method were 9 and 10 cm H_2_O. At zero end-expiratory pressure, the *P*_cond_ method retrieved AOPs of 11 and 10 cm H_2_O, respectively. When the PEEP was increased to 10 cm H_2_O, meaning at or above the AOP, no AOP was detected above the PEEP with the *P*_cond_ method, further validating the proof-of-concept (Fig. 2).

### Performance of P_cond_ method

A total of 213 patients from the DriVV (*n* = 45) and PREMIER (*n* = 168) cohorts were included in the study. Their main characteristics are summarized in Additional file 1: Table E1. According to the standard method, 55 patients (26%) had an AOP above 5 cm H_2_O (the level of PEEP at which the AOP was sought), with a median value of 10 cm H_2_O [9–13].

The performance of *P*_cond_ method for airway closure detection is shown in Table 2. The *P*_cond_ method enabled the detection of airway closure with a sensitivity of 93% and a specificity of 91%. *P*_cond_ method was characterized by a high negative predictive value.

**Table 2:** Performance of the *P*_cond_ method for the detection of airway closure

	*P*_cond_ method
Sensitivity (%)	93
Specificity (%)	91
Positive predicted value (%)	77
Negative predicted value (%)	97
Likelihood ratio of positive test	9.77
Likelihood ratio of negative test	0.08
Diagnostic accuracy (%)	91
Youden index	0.83

*P*_cond_: conductive pressure. See text and Fig. 1 for definition

**Fig. 3:**
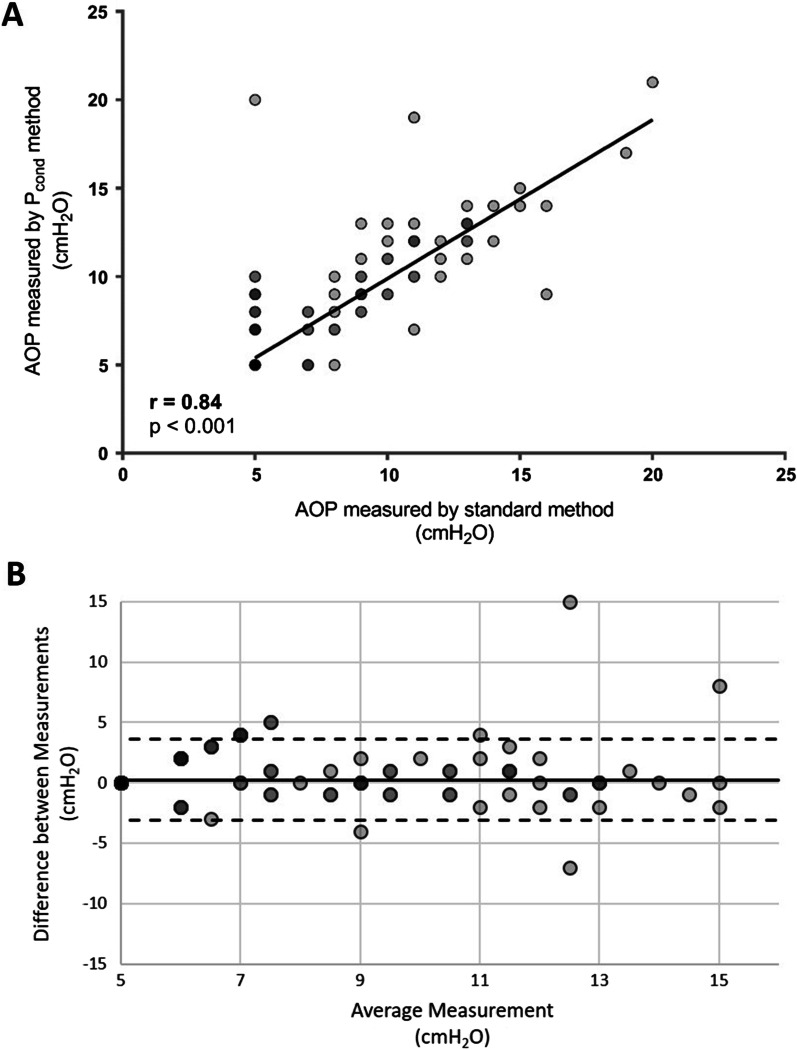
Precision of airway opening pressure measurements using *P*_cond_ methods. **A** Spearman correlation between standard method and *P*_cond_ method. The black line represents the linear regression slope. The gray circles represent individual data. **B** Bland–Altman plots between the standard method and the *P*_cond_ method. The solid black line represents the bias. The dashed black lines represent the upper and lower limits of agreements. Gray circles represent individual data. Note that circles are filled in gray with a certain level of transparency to enhance the visibility of overlapping points, which appear darker

AOP obtained by *P*_cond_ method showed a strong correlation with AOP obtained by standard method (*r* = 0.84, *p* < 0.001, Fig. 3). The Bland–Altman plot for *P*_cond_ method showed a bias of 0 with limits of agreement between − 3 and 4 cm H_2_O (Fig. 3). The median difference between standard and *P*_cond_ methods measurements was 0 cm H_2_O [0–0].

### Clinical study: tolerance assessment

The main characteristics of the 45 patients with assessment of the tolerance of the different AOP measurement methods are shown in Additional file 1: Table E2. Of these, 16 (36%) had an AOP greater than 5 cm H_2_O, with a median value of 8 cm H_2_O [7–12].

**Fig. 4:**
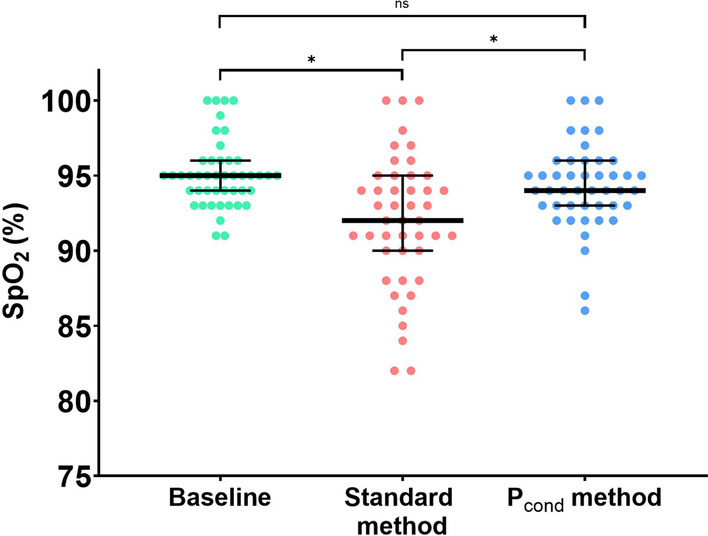
Change in SpO_2_ during ventilator setting adjustments required by airway opening pressure measurement methods. Green circles represent SpO_2_ value at baseline, red and blue circles the minimal SpO2 values recorded during ventilator settings adjustment for standard method and P_cond_ method, respectively. Thick black lines represent the median and thin black lines the interquartile range. *Denotes statistical significance, “ns” indicates non-statistical significance

The SpO_2_ resulting from ventilator’s settings adjustment significantly decreased during the standard method but not during the *P*_cond_ method (Fig. 4). Thus, two (4%) patients experienced a SpO_2_ ≤ 88% versus 10 (22%) during standard method (*p* = 0.013). Additionally, standard method was associated with higher maximal systolic blood pressure (Table 3).

**Table 3:** Respiratory and hemodynamic tolerance of airway opening pressure detection and measurement methods

	Baseline	Standard method	*P*_cond_ method	*p*
PEEP, cm H_2_O	12 [10–12]	5 [5–5]	5 [5–5]	–
SpO_2_, %	95 [94–96]	–	–	–
Minimal SpO_2_, %	–	92 [90–95]	94 [93–96]	< 0.0001
Variation in SpO_2_, %	–	− 2 [− 4 to − 1]	0 [− 1 to 0]	< 0.0001
Respiratory rate, cycles/min	28 [23–30]	5 [5–5]	20 [20–20]	< 0.0001
Heart rate, beats/min	73 [60–91]	–	–	
Minimal heart rate, beats/min	–	73 [60–91]	74 [59–91]	0.36
Maximal heart rate, beats/min	–	73 [62–92]	76 [63–92]	0.47
Systolic arterial pressure, mmHg	117 [104–131]	–	–	–
Maximal systolic arterial pressure, mmHg	–	125 [106–136]	122 [108–136]	0.04
Variation in systolic pressure, mmHg	–	4 [0–17]	1 [− 1 to 15]	0.04
Mean arterial pressure, mmHg	75 [70–87]	–	–	–
Minimal mean arterial pressure, mmHg	–	75 [70–88]	77 [71–84]	0.62
Variation in mean arterial pressure, mmHg	–	0 [− 1 to 4]	0 [− 3 to 5]	0.14

See text and Fig. 1 for methods definitions

## Discussion

The main findings of our study are as follows: time-airway pressure curve analysis during volume assist control ventilation with a constant-flow rate of 60 L/min allowed detection of airway closure and measurement of AOP in passively ventilated patients by subtracting *P*_res_ from *P*_cond_. Noticeably, the AOP assessment was better tolerated with this new method, which does not require the use of low-flow insufflation.

### Conductive pressure definition

The present study is the first to rise the concept of conductive pressure. Until now, the first abrupt change in slope of the airway pressure waveform during volume assist control ventilation at the usual flow rate was considered to entirely be due to the *P*_res_ above the total PEEP [23]. We herein showed that it also depends on the AOP above the total PEEP. This justifies the concept of *P*_cond_, which carries information on *P*_res_, intrinsic PEEP, and AOP above the total PEEP. It is noteworthy that *P*_cond_ method allows to measure a pressure threshold to inflate lung greater than set PEEP, which correspond to AOP in the absence of intrinsic PEEP (as described in the “Methods” section).

### The interest of a new method for the detection and measurement of AOP

Airway closure phenomenon is frequent during ARDS [1], occurring in 23–52% of patients [2–9]. Its detection and measurement of AOP are of crucial importance to adequately assess respiratory mechanics [3] and properly adapt ventilator’s settings. In fact, if neglected, it may lead to overestimation of the driving pressure, underestimation of the respiratory system compliance, and misinterpretation of recruitability [4]. This can significantly interfere with clinical judgment and lead to inappropriate interventions, such as inappropriate ventilator settings or unwarranted adjunctive measures (e.g. excessive sedation). Neglecting the AOP may also have influenced the results of previous studies on the potential relationship between respiratory mechanics and clinical outcomes [24]. Additionally, ventilation with a level of PEEP set below the AOP may generate cyclic opening and closing of small airways that may promote ventilator-induced lung injury [14]. Until now, the assessment of AOP has required low-flow insufflation to make the resistive pressure negligible [1]. One previous report showed that such low-flow insufflation may be poorly tolerated by some patients, with some decrease in PaO_2_ and increase in PaCO_2_ [15]. In this study, we confirmed that it may lead to a significant decrease in oxygenation. The new method of AOP detection and measurement proposed in this study offers the double advantage of requiring less changes in the ventilator settings (in particular no modification of the flow rate) and of being significantly better tolerated by the patients in terms of oxygenation and hemodynamics.

### Performance of P_cond_ method and clinical application

The *P*_cond_ method showed comparable diagnostic performance to the standard method to detect airway closure phenomenon through the detection of AOP. Furthermore, in the case of AOP above the PEEP, the value measured by this new method correlated well with that measured by the standard method. The Bland–Altman analysis of the *P*_cond_ method demonstrated a negligible bias of 0 cm H_2_O, indicating good agreement with the standard method. The limits of agreement ranged from − 3 to 4 cm H_2_O, suggesting moderate precision but acceptable variability within clinical practice. The high negative predictive value (97%) allows at least the *P*_cond_ method to be used to identify the patients in whom the use of the standard low-flow method to search for an AOP is futile. Based on its diagnostic performance, some pragmatic clinical applications of the *P*_cond_ measurement can be proposed. Above all, *P*_cond_ determination may help to identify patients who do not require low-flow insufflation due to the absence of airway closure. In cases where AOP is detected, several strategies can be considered, such as performing low-flow insufflation in such selected patients, increasing the PEEP level until *P*_cond_ equal *P*_res_, or simply relying on the AOP value provided by this new method. These strategies warrant further investigation in future studies. However, it is important to note that all waveforms analyses were conducted offline in the current study. The feasibility of employing the *P*_cond_ method at the bedside should depend on the sample rate at which the ventilator displays the airway pressure waveform on its screen and requires further research. Nevertheless, as we demonstrated that the airway pressure waveform carries information about a possible AOP, one may also hypothesize the feasibility of developing future algorithm to automatically detect and measure the AOP during standard constant-flow ventilation.

### Strengths and limitations of the study

The main strength of our study lies in its bench-to-bedside approach, from the proof-of-concept of the new method to their assessment during clinical application. Furthermore, although assessment of external validity, inter-observer reproducibility and implementation of this new method in the clinical setting will require further studies, the assessment of diagnostic performance in two different cohorts reinforced external validity. Finally, the definition of the *P*_cond_ opens new investigation perspectives in the field of respiratory mechanics.

Our study has several limitations. First, the new method rely on two assumptions: 1—*P*_res_ remains constant during insufflation, which may not be true in all patients; 2—the flow is constant during insufflation, which may depend on the pressurization performance of the ventilator, especially at the beginning of the insufflation. Future algorithms for automatic detection of AOP should take into account the actual flow rate to more accurately calculate the resistive part of the *P*_cond_ and thus better measure potential AOP. Second, *P*_cond_ was measured offline. Applicability of the method in the clinical setting with the use of ventilators’ screens should be assessed in future studies before being encouraged. Particularly, it may be influenced by the ventilator waveforms display rate. Inter-observer reproducibility should also be assessed. Third, the FiO_2_ was kept constant and was not increased to 100% during the tolerance assessment. This may have significantly influenced the results. On the other hand, pure oxygen at a PEEP of 5 cm H_2_O may have promoted derecruitment and altered assessment of respiratory mechanics [25].

## Conclusion

Determination of conductive pressure during constant-flow assist control ventilation may permit to easily detect airway closure and measure AOP without requiring any additional maneuvers.

## Supplementary Information

**Additional file 1**. Additionnal methods and results.

## Data Availability

The datasets used and/or analyzed during the current study are available from the corresponding author on reasonable request.

## Abbreviations

AOP: Airway opening pressure
AOP_sim_: Simulated airway opening pressure
ARDS: Acute respiratory distress syndrome
ASL: Active servo lung
Ers: Respiratory system elastance
HR: Heart rate
Paw: Airway pressure
*P*_cond_: Conductive pressure
PEEP: Positive end-expiratory pressure
PEEPtot: Total positive end-expiratory pressure
*P*_el_: Elastic pressure
*P*_res_: Resistive pressure
RR: Respiratory rate
Rrs: Respiratory system resistance
SpO_2_: Peripheral oxygen saturation
TEC: Thiel embalmed cadavers
