# Supplementary material for: A novel method for assessment of airway opening pressure without the need for low-flow insufflation
Source: Crit Care. 2023 Jul 7;27:273. doi: 10.1186/s13054-023-04560-0 (PMC10329375; doi:10.1186/s13054-023-04560-0)
Supplement: Supplementary file 1 — Additional file 1. Additionnal methods and results. [file 13054_2023_4560_MOESM1_ESM.docx]

**ONLINE DATA SUPPLEMENT**

**A novel method for assessment of airway opening pressure without the need for low-flow insufflation**

Anne-Fleur HAUDEBOURG, Elsa MONCOMBLE, Arnaud LESIMPLE, Flora DELAMAIRE, Bruno LOUIS, Armand MEKONTSO DESSAP, Alain MERCAT, Jean-Christophe RICHARD, François BELONCLE, Guillaume CARTEAUX

**ADDITIONAL METHODS**

## **Proof-of-concept evaluation: Bench study**

*Mechanical bench:*

We used an Active Servo Lung 5000 test lung (ASL5000®; IngMar Medical, Pittsburg, PA, USA). First, we simulated a patient model with an airway opening pressure of 10 cm H_2_O using a model with a nonlinear compliance: the first compliance was near zero (set to 0.1 mL/cm H_2_O) to simulate complete airway closure until an airway pressure of 10 cm H_2_O was reached, the second compliance above 10 cm H_2_O was set to 40 mL/cm H_2_O. Second, we simulated two controls: 1- no airway closure by using the two-compartment model with a linear compliance of 40 mL/cm H_2_O; 2- no airway closure but with a nonlinear compliance, as described in some ARDS patients (19), with a lower inflection point at 10 cm H_2_O: the first compliance (also called starting compliance) was set to 20 mL/cm H_2_O and the second compliance (also called inflation compliance) to 40 mL/cm H_2_O. Airway resistance was set to 10 cm H_2_O/L/sec for all conditions.

Thus, as described in the “Non-Linear Compliance Editor” section of the user’s manual of ASL 5000 (<https://www.ingmarmed.com/wp-content/uploads/2016/11/80-31-760-ASL-5000-Users-Manualpv1-1.pdf>), the model was configured with the following parameters to simulate an airway opening pressure of 10 cm H_2_O:

|  | P (cmH_2_O) | V(L) |
| --- | --- | --- |
| Left End Point | 0.0 | 0.000 |
| Inflection Point 1 | 10.0 | 0.001 |
| Y Intercept 1 | 0.0 | 0.002 |
| Y Intercept 2 | 0.0 | 1.200 |
| Inflection Point 2 | 40.0 | 1.201 |
| Right End Point | 60.0 | 2.001 |

For the second control (no airway closure but non-linear compliance), we configured the following parameters:

|  | P (cmH_2_O) | V(L) |
| --- | --- | --- |
| Left End Point | 0.0 | 0.000 |
| Inflection Point 1 | 10.0 | 0.200 |
| Y Intercept 1 | 0.0 | 0.201 |
| Y Intercept 2 | 0.0 | 1.399 |
| Inflection Point 2 | 40.0 | 1.400 |
| Right End Point | 60.0 | 2.200 |

Volume assist control ventilation with constant flow was applied to the three models using a Puritan Bennett™ 980 ventilator (PB 980; Medtronic, Dublin, Ireland). The tidal volume was set to 420 mL (6 mL/kg of predicted body weight (PBW) for an “average male patient” of 175 cm of height). For reference standard method application, low-flow insufflation was performed at a constant flow rate of 5 L/min, a PEEP of 5 and 12 cmH_2_O and a respiratory rate (RR) of 5 cycles /min. P_cond_ method was assessed at a constant flow rate of 60 L/min, a PEEP of 5 and 12 cm H_2_O and a RR of 20 cycles /min.

*Physiological bench:*

We assessed the proof-of-concept of the new AOP measurement methods in two Thiel embalmed Cadavers (TEC) in whom an AOP ≤ 10 cm H_2_O was detected using the reference standard method. TEC are human corpses embalmed after a method described by Walter Thiel (20,21), whose aspect is close to the living anatomy and with preserved elasticity and flexibility. The corpses were used from a specific donation program of the anatomy laboratory of UQTR (Université du Québec à Trois-Rivières) and experiments were conducted in accordance with Canadian regulation and approved by the ethics committee of the
UQTR (SCELERA-19-01-PR02). TEC were intubated and mechanically ventilated with a Monnal T60 ventilator (Air Liquide Medical Systems, Antony, France). After ventilation and recruitment period, suctioning was performed with a single-use catheter via the endotracheal tube in order to remove the preservation fluid from the airways and lung. A systematic chest x-ray was performed in order to validate endotracheal tube proper positioning and to verify proper lung aeration. Volume assist-control ventilation with constant flow was applied with a tidal volume of 6 mL/kg of predicted body weight. Standard ventilation was applied with a constant flow rate of 60 L/min, a PEEP of zero and 10 cm H_2_O and a RR of 18 cycles /min. Low-flow insufflation was performed with a constant flow rate of 5 L/min, a PEEP of zero and 10 cm H_2_O and a RR of 5 cycles /min.

***Performance of the new method: physiological study***

We assessed the performance of the new method for both detection of airway closure and measurement of AOP in two prospective observational cohorts collecting detailed data on respiratory mechanics in patients under invasive mechanical ventilation: the DriVV and the PREMIER cohort. The DriVV cohort (2018-A00867-48), conducted at the medical ICU of the Henri Mondor University Hospital, Créteil, France and approved by the “CPP Sud-Ouest et Outre Mer III” ethics committee, prospectively included patients with ARDS according to the Berlin definition (3). Non-inclusion criteria were the followings: age < 18 years, pregnancy, the need for strict control of PaCO_2_ (e.g., severe neurological impairment) and patients under legal protection.

The PREMIER cohort (2017-A02842-51) conducted in the medical ICUs of the University Hospital of Angers and the University Hospital of Strasbourg, France and approved by “CPP Sud-Est I” ethics committee, prospectively included patients on invasive ventilation fed by gastric tube. Non-inclusion criteria were the followings: age < 18 years, contraindications to the insertion of a nasogastric tube (esophageal varices, recent esophageal surgery or trauma), pregnant or breastfeeding woman, patients under legal protection and persons not affiliated or not benefiting from a social security system. In accordance with French law, non-opposition to participate in the study from patients or their next of kin was obtained prior to inclusion in each study.

***Measurements***

For the DriVV cohort, flow and airway pressure were recorded using a heated pneumotachograph AC137A-1 (Biopac Systems, Goleta, CA, USA) connected to a differential pressure transducer TSD160A (Biopac Systems, Goleta, CA, USA) and a differential pressure transducer TSD160C ((Biopac Systems, Goleta, CA, USA) respectively, inserted between the Y piece of the ventilator circuit and the ASL inlet for the mechanical bench study or the endotracheal tube for the physiological bench and DriVV Cohort. Signals were recorded at 1000 Hz using an analog/numeric data-acquisition system (MP 150; Biopac Systems, Goleta, CA, USA). For the PREMIER Cohort, flow, airway pressure, and esophageal pressure-time curves were recorded using a dedicated computer connected to an Engström® or R860® ventilator (GE Healthcare, Madison, WI, USA) at 25 Hz (40 ms sampling time) for offline analysis. To obtain sufficient accuracy in order to detect Pcond, raw data was resampled at 75 Hz (13 ms sampling time) using linear interpolation.

All signals were stored in a computer for subsequent analysis using the Aqcknowledge software (v5.4).

## **Statistics**

Data were analyzed using GraphPad Prism 8.0.1 (San Diego, CA, USA) and SPSS Base 29.0 statistical software package (SPSS, Chicago, IL). Continuous data were expressed as medians (25^th^ – 75^th^ percentiles) and compared using Mann-Whitney test for independent variables and Friedman then Wilcoxon signed rank test for related variables. A Bonferroni correction was applied in case of multiple comparisons. Categorical variables, expressed as percentages, were evaluated using Chi-square or Fisher exact tests as appropriate. A p < 0.05 was considered significant. Standard formulas were used to calculate the sensitivity (true positive / [true positive + false negative]), specificity (true negative / [true negative + false positive]), positive predictive value (true positives / [true positives + false positives]), negative predictive value (true negatives / true negatives + false negatives)), positive likelihood ratio (sensitivity / [1 − specificity]), negative likelihood ratio ([1 − sensitivity] / specificity), diagnostic accuracy ([true positive + true negative] / [true positive + true negative + false positive + false negative]), and Youden index (sensitivity + specificity − 1). Linear correlation analysis was performed to assess whether relationships existed between the reference standard method and new method. Spearman correlation coefficients (r) and uncorrected p-values are presented. Bland–Altman analyses were performed to evaluate agreement between P_cond_ method and reference standard method (20). Using the Bland–Altman methods, the mean differences between both measurements and the 95% limits of agreement, defined as the mean differences ±1.96* standard deviation, were calculated.

**ADDITIONAL RESULTS**

**Table E1:** **Characteristics of patients included in the physiological study**

|  | All patients  (n=213) | Patients with AOP > 5 cm H_2_O  (n=55) | Patients without AOP  (n=158) | p |
| --- | --- | --- | --- | --- |
| Age, years | 64 [54 – 74] | 64 [55 – 72] | 64 [54 – 75] | 0.41 |
| Sex ratio, M/W | 141/68 | 34/19 | 107/49 |  |
| BMI, kg/m^2^ | 26 [23 – 31] | 32 [27 – 40] | 25 [22 – 29] | <0.0001 |
| PaO_2_/FiO_2_, mm Hg | 175 [112 – 243] | 153 [104 – 239] | 178 [119 – 250] | 0.13 |
| Airway resistance, cmH_2_O/L/sec | 17 [14 – 20] | 21 [17 – 23] | 16 [14 – 19] | <0.0001 |
| Respiratory system compliance, mL/cmH_2_O | 45 [35 – 56 ] | 40 [30 – 53] | 47 [36 – 57] | 0.03 |

*BMI: Body Mass Index; AOP: Airway Opening Pressure.*

**Table E2: Characteristics of patients included in the clinical part of the study for tolerance assessment**

|  | **All patients (n=45)** |
| --- | --- |
| Age, years | 61 [54 – 68] |
| Sex ratio, M/W | 34/11 |
| BMI, kg/m^2^ | 27 [23 – 33] |
| Covid-19 related ARDS | 26 (58%) |
| MV duration before inclusion, days | 2 [0.5 – 4] |
| PaO_2_/FiO_2_, mm Hg | 119 [89 – 170] |
| Neuromuscular blocking agents | 45 (100%) |
| Nitric Oxid (NO) | 7 (16%) |
| Position during measurements  Supine  Prone | 40 (89%)  5 (11%) |
| Ventilator parameters at baseline  FiO2, %  Tidal volume, mL  Tidal volume, mL/kg IBW  Respiratory rate, cycles/min  PEEP, cmH_2_O  Flow rate, L/min | 60 [50 – 70]  400 [350 – 445]  6 [5.8 – 6.1]  27 [23 – 30]  12 [10 – 13]  60 [60 – 60] |
| AOP > 5 cmH_2_O, n (%) | 16 (36%) |
| AOP, cmH_2_O *(n=16)* | 8 [7 – 12] |
| Airway resistance, cmH_2_O/L/sec | 18 [15 – 20] |
| Respiratory system compliance, mL/cmH_2_O | 36 [29 – 50] |
| R/I ratio | 0.27 [0.09 – 0.40] |
| Intrinsic PEEP, cmH_2_O | 0 [0 – 1] |

*BMI: Body Mass Index; MV: mechanical ventilation; ARDS: Acute Respiratory Distress Syndrome; PEEP: positive end-expiratory pressure; AOP: Airway Opening Pressure, R/I: recruitment to inflation ratio.*
